# Industrial Internet of Things-Based Collaborative Sensing Intelligence: Framework and Research Challenges

**DOI:** 10.3390/s16020215

**Published:** 2016-02-06

**Authors:** Yuanfang Chen, Gyu Myoung Lee, Lei Shu, Noel Crespi

**Affiliations:** 1 Institut Mines-Télécom, Télécom SudParis, Evry 91011, France; yuanfang_chen@ieee.org (Y.C.); noel.crespi@mines-telecom.fr (N.C.); 2 Liverpool John Moores University, Liverpool L3 3AF, UK; g.m.lee@ljmu.ac.uk; 3 Guangdong University of Petrochemical Technology, Maoming 525000, China

**Keywords:** big data analytics, collaborative intelligence, industrial sensing intelligence, Internet of Things

## Abstract

The development of an efficient and cost-effective solution to solve a complex problem (e.g., dynamic detection of toxic gases) is an important research issue in the industrial applications of the Internet of Things (IoT). An industrial intelligent ecosystem enables the collection of massive data from the various devices (e.g., sensor-embedded wireless devices) dynamically collaborating with humans. Effectively collaborative analytics based on the collected massive data from humans and devices is quite essential to improve the efficiency of industrial production/service. In this study, we propose a collaborative sensing intelligence (CSI) framework, combining collaborative intelligence and industrial sensing intelligence. The proposed CSI facilitates the cooperativity of analytics with integrating massive spatio-temporal data from different sources and time points. To deploy the CSI for achieving intelligent and efficient industrial production/service, the key challenges and open issues are discussed, as well.

## 1. Introduction

Given the rapidly-evolving demands of industrial production/service for safety [1,2], efficiency [3] and environmental friendliness [4], various sensors and wireless devices have been widely deployed to industrial environments [5,6]. On this basis, the Internet of Things (IoT) for industrial applications is being gradually developed [7,8], which is named the Industrial IoT (IIoT) [9]. With the IIoT, massive data are being collected on a daily basis. Collaboratively analysing based on the massive data that come from different objects and different time points can help to obtain efficient and cost-effective solutions to achieve safe, highly efficient and eco-friendly industrial production/service [10]. Moreover, such data-centric solutions are flexible and low cost.

In this study, based on the massive spatio-temporal data from different devices and different time points, with developing the potential of big data analytics, we design a collaborative sensing intelligence (CSI) framework. This framework facilitates the cooperativity of big data analytics.

**Figure 1:**
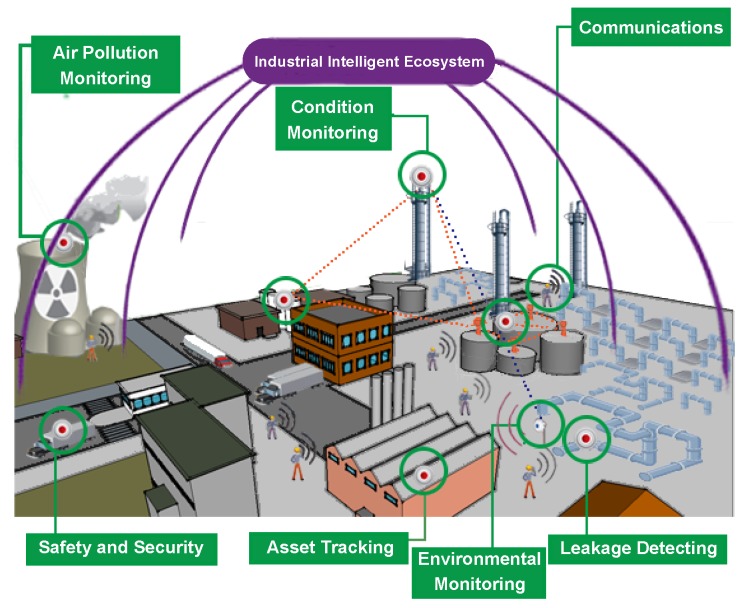
An industrial intelligence ecosystem. In this ecosystem, different objects (e.g., humans and machines) are working as an efficient whole with effective dynamic collaboration. The ecosystem consists of two parts: (i) sensing of humans with smart devices; humans (workers) share information with each other and with various sensors; and (ii) sensing of sensors embedded in machines. Through the sensors that are embedded in different industrial equipment, a variety of status information (even weather information) can be obtained and shared with other information sources.
- This study clearly answers why and how designing the CSI framework based on the IIoT can be achieved. The key components of this framework are described in detail. Moreover, two on-going efforts about developing the framework are introduced and discussed. This CSI framework aims to achieve the dynamic collaboration between different objects, and such a collaboration is based on massive spatio-temporal data.
- We list and analyse the challenges and open research issues for developing and realizing the CSI framework.

On the CSI framework basis, an industrial intelligence ecosystem can be constructed with the dynamic collaboration of different objects (an example is illustrated in Figure 1).

The scientific contributions of this article are listed as follows.

- The definitions of both terms, collaborative intelligence (CI) and industrial sensing intelligence (ISI), are proposed under the background of IoT and big data analytics.

The remainder of this article is organized as follows. In Section 2, we clearly define the terms CI and ISI and discuss their advances. Section 3 answers why and how we design the CSI framework, with integrating CI into ISI. Moreover, this section also displays and describes the key components of CSI framework. On this basis, two on-going efforts are introduced and discussed to provide the details about how to achieve CSI in industrial applications. Section 4 presents what are the challenges and open research issues for deploying this CSI framework to the dynamic environment of industry. This article is concluded in Section 5.

## 2. Definitions and Advances

As the basis of the CSI framework, the terms CI and ISI are clearly defined, and their advances are discussed in this section.

### 2.1. What is Collaborative Intelligence

In industrial production/service, based on the IIoT: (i) What is intelligence? (ii) Why do we need this intelligence? (iii) What is and why do we need “collaboration”? Then, from the answers to these questions, the term CI can be clearly defined.

The intelligence of industrial production/service in the IIoT can be described as: industrial production/service includes a series of complex and dangerous processes, so how to minimize the manual intervention in these processes is an important issue for improving the safety, efficiency and eco-friendliness of production/service. On this basis, automation becomes very important [11]. Then, the intelligence can be defined as the ability to acquire information or knowledge based on the IIoT and applying the acquired information or knowledge to construct deliverable process models, for achieving or improving the automation of industrial production/service.

From the above description about intelligence, the development of the intelligence on processes is a requisite and important step to realize the high efficiency automation of industrial production/service.

In addition, effective collaboration between different industrial processes is important and necessary to realize the intelligence of industrial production/service. That is, the intelligence on industrial production/service is a series of collaborations between different industrial processes.

The definition of “collaborative intelligence” is described in Definition 1.

**Definition 1.**  Under the background of big data analytics, collaborative intelligence is the ability to acquire information or knowledge from massive data, for constructing a problem-solving network (A problem-solving network is proposed for exploiting the potential of “the collaboration between different objects” and “the wisdom of crowds”, and for transferring information-intensive organizations to a network society. It is set for solving problems rather than building relationships). Based on the network, the purpose of collaborative intelligence is to realize the automation of industrial production/service or to improve the performance of the automation. Moreover, the massive data are collected from different autonomous equipment of industrial systems.

In summary, due to the close correlation between processes, the collaboration between them is indispensable. With analysing the massive data that come from different autonomous equipment, the collaboration can be achieved. Based on such a collaboration, intelligence can be easily and quickly deployed on different industrial systems. Along with this deployment, the automation of industrial production/service can be developed. Moreover, as the basis of intelligence, acquiring information or knowledge is possible based on the massive data that are collected by various sensors and wireless devices. These sensors and wireless devices are embedded in autonomous equipment, for monitoring or controlling the processes of industrial production/service.

### 2.2. What is Industrial Sensing Intelligence

Based on the sensors and wireless devices deployed in industrial environments, the definition of “industrial sensing intelligence (ISI)” is described in Definition 2. This definition considers the characteristics of industrial problems (the characteristics of a typical industrial problem include these two aspects: (i) the environment of industrial production/service is highly dynamic and complex [12]; and (ii) industrial production/service includes a series of highly-correlated processes [13]) and is under the background of big data analytics.

**Definition 2.**  Through dynamically mining and analysing the massive spatio-temporal data that are collected from industrial ecosystems (Figure 1), useful information/knowledge can be acquired to improve the ability of industrial automation.

Definition 2 has taken into account these three important aspects:

- Mining and analysing spatio-temporal data: The data are collected from industrial ecosystems (an example is shown in Figure 1). In such ecosystems, there are various sensors and wireless devices to sense surroundings and to collect the data from different data sources and time points. Based on the collected data, mining and analysing the data have a certain logic.
- Acquiring useful information/knowledge: This is the important aspect to achieve the “intelligence” of industry. Industrial automation is the first step of realizing industrial intelligence. With acquired useful information/knowledge, industrial automation can be improved and enter into the intelligent era.
- Considering the characteristics of industrial problems: In the definition, the description, “through dynamically mining and analysing”, is to considered the characteristic about “highly dynamic and complex”, and the description, “spatio-temporal data”, is to considered the characteristic about “a series of correlated processes”.

From the definition of ISI, it is obvious that ISI consists of physical sensing, data mining and analysis, as well as information/knowledge acquirement and utilization.

### 2.3. Advances

#### 2.3.1. Collaborative Intelligence

CI is able to utilize extensive information or knowledge to construct a problem-solving network, for complex industrial problems. Based on this, collaborative intelligent systems are built for complex industrial production/service.

CI involves extensive and intensive collaboration of different members as an efficient team to solve problems. Such collaboration possesses great potential on problem resolution under challenging environments [14]. It obviously can provide more information/knowledge for designing improved solutions than any single member could. It achieves the flexibility of how members are deployed. It gives a non-stop real-time learning opportunity to a team. Moreover, such collaboration has the potential of integrating diverse contributions (different members contribute different information/knowledge, skill and experience to a problem resolution) into a platform to produce a creative solution for successfully solving a problem [15].

Based on the above advantages, CI has been widely studied. As an important existing platform for CI, HUB -CI (HUB with CI) [16] is the next generation of collaboration-supported system developed at Purdue University. On this platform basis, Prabhu Devadasan *et al.* have designed the model CIMK that measures CI by the multi-objective optimization on the parameters of collaboration and suggest the optimal operating points for various clients, with greater flexibility.

The advance of CI is briefly discussed. Relevant studies are classified in Table 1, and we list some typical literature for each classification as examples. Additionally, we discuss several studies in detail to make the meaning of each classification easy to be understood.

**Table 1:** Classification of the studies on collaborative intelligence (CI).

Classification	Typical Application	Typical Recent Literature
Human-based CI	Smart search and recommendation in social networks	[17,18,19,20,21,22,23]
IoT-based CI	Optimizing the performance of intelligent systems	[24,25,26]

In Table 1, the relevant studies can be classified into two classes, human-based CI and IoT-based CI, depending on the difference of the participants.

**Human-based CI.** As the typical applications of human-based CI, the smart search and recommendation of social networks have been widely studied.

In the literature [17,18], Vincent W. Zheng *et al.* have developed a mobile recommendation system to answer two popular location-related queries in our daily life: “(1) If we want to do something such as sightseeing or dining in a large city like Beijing, where should we go? (2) If we want to visit a place such as the Bird’s Nest in Beijing Olympic park, what can we do there?”. This system includes three important algorithms that are based on collaborative filtering to address the data sparsity problem (the data comes from each user and is used to make the recommendation, but each user has limited data, which makes the recommendation task difficult). By these three algorithms, the advantages of collaboration can be highlighted. The first algorithm uses a collective matrix factorization model to provide a recommendation, based on the merged data from all of the users. The second algorithm uses a collective tensor and matrix factorization model to provide a personalized recommendation. The third algorithm further improves the previous two algorithms by using a ranking-based collective tensor and matrix factorization model.

As the important supportive work of the above-mentioned achievement from Vincent Zheng *et al.*, in the literature [19], they have presented user-centred collaborative location and activity filtering (UCLAF) to merge the data from different users together and have applied the collaborative filtering to find like-minded users and like-patterned activities at different locations.

**IoT-based CI.** As the typical application for such CI, optimizing the performance of intelligent systems has attracted attention.

In IoT and intelligent system-related studies, the study of intelligent transportation systems is an important aspect. In the literature [24], a collaborative framework is proposed for the real-time traffic information collection, fusion and sharing. The real-time traffic information is reported by various front-end devices of intelligent transportation systems, for example a vehicle-mounted GPS receiver. The framework integrates real-time traffic information from different data sources to be able to improve the performance of the intelligent transportation system, for example enabling the high-accuracy prediction for real-time traffic status.

As another important intelligent system, the intelligent healthcare service system, Byung Mun Lee *et al.* have introduced a collaboration protocol to share health information between IoT personal health devices [25]. By such information sharing, the quality of healthcare service can be improved.

On the other side, the collaboration between different members perhaps results in serious mistakes. If a collaboration is not efficient and even incongruous, a minor mistake in this collaboration will fall into a syndrome known as “groupthink” [27], and the syndrome causes the mistake to be amplified, which results in a fiasco [28]. How to make a collaboration efficient is an important and difficult problem. The book [29] presents an approach. Its premise is that preliminary work is performed by professionals of the intelligence community: mining information/discovering knowledge from the target work and members of a collaborative team. The effectiveness and correctness about this premise have been verified in the research achievement [30].

#### 2.3.2. Industrial Sensing Intelligence

Based on the development of IoT technology in industrial applications, sensing intelligence has drawn wide attention, on account of these advantages: (i) with the help of sensing intelligence, efficient monitoring and controlling can be achieved to reduce the costs and energy consumption of industrial production/service; and (ii) with the help of sensors and wireless devices embedded in industrial machines and systems, the maintenance of these machines and systems is controllable and automatable; especially, these machines and systems are deployed in remote and hard-to-reach areas. Sensing intelligence has been successfully applied to many industrial applications, such as monitoring, controlling, maintenance and security [31]. Typical industrial applications of sensing intelligence are introduced as follows.

**Factory automation.** A factory is a highly dynamic ecosystem, so automation is necessary in such an environment. Traditional actuators combined with control units have been used for factory automation. With the development of wireless and sensor technologies, the adoption of WSNs (wireless sensor networks) and RFID (radio frequency identification) on the actuators and control units for factory automation has experienced impressive growth over the past decade [32,33]. This is ISI-based factory automation.

In the manufacturing environment of a factory, two main activities are included, manufacturing operations and equipment maintenance [34]. In recent years, based on these two main activities, the studies on factory automation pay much attention to these four aspects [6,35]: (i) the monitoring and controlling for manufacturing processes; (ii) the safety and maintenance for machines; (iii) the resource tracking for manufacturing workshops; and (iv) high-level logistics and supply chain management.

An ISI-based factory automation system consists of various devices, e.g., sensors, controllers and heterogeneous machines, and these devices can be combined together through the communications between each other. The communication component is the most important part of factory automation. In Table 2, we list the communication protocols that can be used in ISI-based factory automation.

**Table 2:** Relevant protocols for industrial sensing intelligence (ISI)-based factory automation.

Wireless Communication Protocol	Relevant Standard	Maximum Data Rate (Mbit/s)	Maximum Data Payload (Bytes)
Bluetooth	IEEE 802.15.1	1	339
Ultra-Wideband (UWB)	IEEE 802.15.3	110	2044
ZigBee	IEEE 802.15.4	0.25	102
WiFi	IEEE 802.11a/b/g	54/11/54	2312

By using ISI-based factory automation: (i) the theoretical study achievements on factory automation can be improved; and (ii) the ability of factory automation can be enhanced to achieve safe, efficient and eco-friendly factory production.

**Energy industry.** As another important application of sensing intelligence, the application environment of the energy industry and factory automation is different. In the energy industry, the sensing intelligence is mainly applied to inaccessible environments to monitor and control industrial systems. In factory automation, the sensing intelligence is mainly applied to highly dynamic and large-scale environments.

With the development of sensing technology and the extensive deployment of sensors, the sensing intelligence-supportive renewable energy industry (e.g., solar, tidal and geothermal energy) has become a new and important study aspect. The equipment for accessing renewable energy is often located in remote areas, such as mountains, seas and volcanoes. Despite this, real-time control is necessary for the units of energy harvesting; for example, for a wind turbine, based on the data from wind-direction sensors, a yaw-drive motor turns the nacelle to face the wind. Moreover, the sophisticated units that are embedded in equipment require frequent maintenance [36]. Sensing intelligence is proposed for both purposes, real-time control and maintenance, in the renewable energy industry [37].

- Real-time control: Based on the development of sensing intelligence in real-time control, first, the real-time data of environmental conditions (environmental conditions include wind speed, temperature, humidity, rainfall and geothermal activity) can be collected by the spatially-distributed sensors and wireless devices. These sensors and wireless devices are embedded in energy-harvesting systems. Then, by using the collected environmental data, the relationship between generated energy and different seasons can be analysed. With the analysed results, the optimal parameter configuration can be acquired and used to control the equipment that is the main component of the energy-harvesting system. In a word, based on sensing intelligence, the process of energy harvesting is highly efficient and automatic [38,39]. Moreover, such real-time intelligent control has been used in smart home services, as well [40].
- Maintenance: The sensors that are embedded in various units of equipment interact with the equipment to take a number of measures, such as the scheduling of maintenance [41], the reconfiguration of certain operations [42] and the emergency shutdown of equipment [43]. With the sensing intelligence in maintenance, unnecessary downtime can be prevented, and equipment failure costs can be reduced.

In recent years, as the important part of the energy industry, the “smart grid” has attracted great attention of researchers. The smart grid represents a vision of the future electricity grid, and it is radically different from current electricity grids that have been deployed. It is an electricity grid that uses analogue or digital communication technology to collect information and take action for automatically improving the efficiency, reliability, economic benefit and sustainability of the production and distribution of electricity [44]. In the literature [45], Ramchurn *et al.* have presented delivering the decentralized, autonomous and intelligent system, the smart grid, as a grand challenge for computer science and artificial intelligence research. As a typical case that is tightly related to the CSI framework in the smart grid, optimizing the electricity usage of electric vehicles is worth studying. For example, with analysing the spatio-temporal trajectory data from an intelligent transportation system, the routing pattern of electric vehicles can be acquired, and then, a national electric supply company can make time- and area-divisory electricity prices to control the usage of electricity and, therefore, to improve the efficiency of the smart grid.

## 3. Collaborative Sensing Intelligence

Based on the above two definitions and IIoT, with integrating CI into ISI, an effective CSI framework can be designed.

### 3.1. Why and How Do We Design the CSI Framework

**Why do we design the CSI framework?** This framework can improve the efficiency of IIoT. In industrial production/service, the internal logical processes are intricate and precise [46]. A large amount of different equipment is involved in these logical processes. For achieving high efficiency industrial production/service, effective collaboration is necessary between different equipment and between different logical processes. The CSI framework can organize multi-sourced data and make different data sources collaborative with each other based on the data. The multi-sourced data are collected from the different equipment and different logical processes of industrial production/service based on the IIoT.

Effective collaboration is possible, with the help of massive data. First, with the application of IoT technology in industry, massive data can be collected by various widely-distributed sensors and wireless devices [7]. Then, as the natural advantage of data, different data are easily processed and even merged together [47]. Finally, the effective collaboration between different equipment and processes can be achieved, with processing and merging different data from multiple sources.

Based on (i) the requirements of industrial production/service and the benefit of CSI framework and (ii) the feasibility of achieving collaboration, the question of “why” is answered.

Moreover, the data-based collaboration can cost-effectively develop the intelligence of industrial production/service [48]. For example, in the chemical industry, different equipment is used in different production stages, and different data are collected. For improving the ability of acquiring information or knowledge and applying the acquired information or knowledge to realize the automation of production, the different equipment collaborating based on the data is an effective and low-cost method.

**Figure 2:**
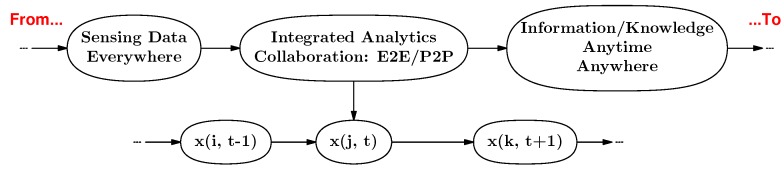
Architecture of the collaborative sensing intelligence (CSI) framework. From the massive data from industrial ecosystems, to mined information/discovered knowledge, which can be used in algorithm design to solve industrial problems. [...,x(i,t−1),x(j,t),x(k,t+1),...] is the state series relating to “location” and “time”, which is used in the collaborative analysis that is based on different state data from different equipment. E2E denotes equipment-to-equipment collaboration, and P2P is for person-to-person.

**How do we design the CSI framework?** Considering the characteristics of industrial problems, integrating CI into ISI is a practicable method to achieve the CSI framework. Various sensors and wireless devices have been widely deployed to industrial equipment, and massive data are collected by these sensors and wireless devices. On this basis, the CSI framework is designed. Figure 2 illustrates the architecture of the CSI framework.

In Figure 2, the availability of massive data is not a problem in industry, owing to the wide deployment of sensors and wireless devices. The problems are: how to integrate different data and filter out noise to find the data we need and how to get the data into the right hands to discover useful information/knowledge. CI empowers systems to intelligently transform vast amounts of operational data into actionable information/knowledge that is accessible and available anytime, anywhere.

Based on the available data from different autonomous equipment of industrial systems, how to construct a problem-solving network is an important and difficult problem, and constructing the problem-solving network is the main target and contribution of CSI, as well. As the common and important features of the data collected from different autonomous equipment, “time” and “location” can be used as collaborative parameters to integrate the different data. A time or location series can be considered as a Markov chain. With the change of time or location, the state of a problem we want to solve undergoes transitions from one state to another in a state space, and the state space includes various current states from different relevant equipment. With the help of the feature parameters of data, the data can be integrated to achieve the collaboration of different autonomous equipment, and the integrated data can be used to mine and discover useful and actionable information/knowledge. On this basis, the problem-solving network can be constructed.

### 3.2. Key Components of CSI

The CSI framework consists of three components (Figure 2): (i) sensing data collection; (ii) integrated analytics with collaboration; and (iii) information mining and knowledge discovery.

**Sensing data collection.** In an industrial ecosystem, massive data have been collecting by the sensors and wireless devices, which are deployed everywhere. Moreover, this component is the basis of integrated analytics, so collecting enough spatio-temporal data is important and necessary.

**Integrated analytics.** This is the core component of CSI. Effective integration of different data is an important and basic premise to mine/discover useful and actionable information/knowledge. Such integration is collaboration-based. How to make different objects collaborate with each other is the problem we need to solve to make the second component more practical. Industrial production/service includes a series of processes and actions, and these processes and actions are location- and time-related. A spatio-temporal Markov chain can be used to process the relationships between these processes and actions. Based on such processing, the collaboration between different objects is achieved.

**Figure 3:**
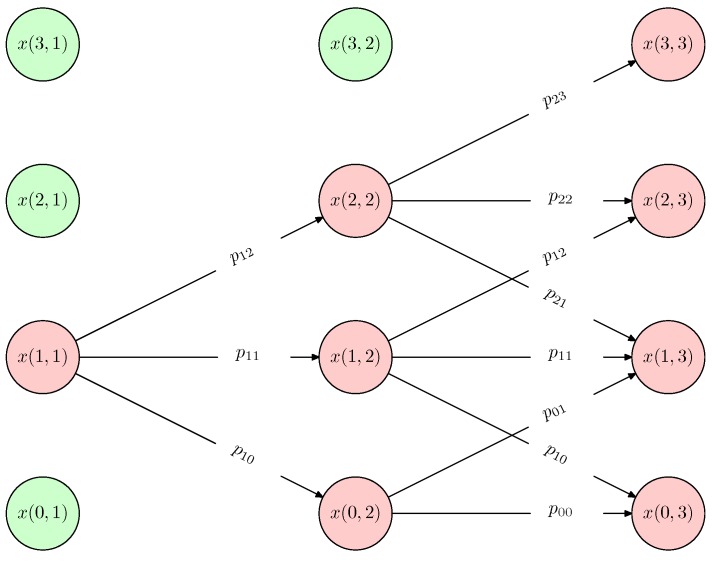
A spatio-temporal Markov chain for the processes of industrial production/service. $P=\{p_{10},...,p_{ij},...\}$ (i,j∈{0,1,2,3}) is the set of processes; x(i,t) (i∈{0,1,2,3},t∈{1,2,3}) denotes the state space at the time *t*; and *i* is the location number of the equipment that has the state x(i,t).

The detailed design and description of a spatio-temporal Markov chain [49,50] are shown as follows. First, a series of processes and actions of industrial production/service produces a series of different states, ...,x(i,t−1),x(j,t),x(k,t+1),..., where x(.,.) is the function of the parameters “location” and “time”. Then, these states meet the Markov property that is described in Definition 3. Finally, the state transitions of industrial processes can be denoted by a spatio-temporal Markov chain, and the state transitions are based on the state space of industrial production/service (an example is illustrated in Figure 3).

**Definition 3.**  A stochastic process has the Markov property, if the conditional probability distribution of future states of the process depends only on the current state, not on a series of preceding states. Therefore, the Markov property can be formulated as: let {X(t),t≥0} be a time continuous stochastic process, which is assumed to be the set of non-negative integers, and then for every n≥0, time points $0\leq t_{0}<t_{1}<\cdot \cdot \cdot <t_{n}$, and states $x_{0},x_{1},...,x_{n}$, the process holds that $P\left(X\left(t_{n}\right)=x_{n}|X\left(t_{n-1}\right)=x_{n-1},X\left(t_{n-2}\right)=x_{n-2},...,X\left(t_{0}\right)=x_{0}\right)=P\left(X\left(t_{n}\right)=x_{n}|X\left(t_{n-1}\right)=x_{n-1}\right).$

This definition shows that only the current state provides information to the future behaviour of the process. Historical states of the process do not add any new information.

Figure 3 provides an example to explain how to do data processing by the spatio-temporal Markov chain.

The spatio-temporal data of this example are a series of states (x(i,t)), and the states at different time points are linked by a set of processes ($p_{ij}$). As the most important information that can be used to link two different states, the location and time stamp are included in each state. In this example, there are four states in the state space of the time point t=1, x(0,1),x(1,1),x(2,1),x(3,1). The state x(1,1) transfers to x(0,2),x(1,2),x(2,2), with corresponding processes $p_{10},p_{11},p_{12}$, and these transitions are based on certain probabilities. As time goes on, step by step, the Markov chain of this specific industrial production/service can be achieved. Such a Markov chain enables the collaboration between different things and time points, based on the massive spatio-temporal data.

**Information mining and knowledge discovery.** On the second component basis, with the help of (i) the representative parameters of industrial processes and (ii) the spatio-temporal Markov chain that is based on the representative parameters, the rules about the industrial processes can be learned, and then, these rules form useful and actionable information/knowledge according to a particular logical sequence. Based on the mined information and the discovered knowledge, various intelligent algorithms can be designed to solve the problems and to meet the requirements of industry.

### 3.3. On-Going Efforts

The CSI framework simplifies the integrated analytics between different data sources and integrates these data sources with their respective semantics, to enable an industrial problem to obtain an optimized solution with using comprehensive information. Based on two on-going efforts, the details of developing the CSI framework in industrial applications are visually provided.

#### 3.3.1. Dynamic Detection of Toxic Gases

As an important part of industry, in large-scale petrochemical plants, the leakage of toxic gases is a serious threat to humans and the environment [51]. It is necessary to develop an intelligent leakage detection solution for timely rescue and control.

The industrial production of large-scale petrochemical plants can be represented by a series of collaborative behaviours in dynamic environments. However, in most existing large-scale petrochemical plants of China, for instance, only static wireless sensor nodes are deployed for detecting toxic gases. These static nodes are independent of each other to alert operators to the possible leakage of toxic gases. A static node raises the alarm, when and only when the sensed reading for a certain toxic gas is larger than a predefined threshold in a certain location. Because of these three “certainties”, (i) certain toxic gas, (ii) predefined threshold and (iii) certain location, the static sensor-based detecting systems are at a distinct disadvantage in dynamic industrial production environments. This “disadvantage” includes four aspects:

- It is difficult to locate the leak source of a toxic gas without tracking the change of concentration of the toxic gas. The concentration of a toxic gas is constantly changing as locations shift and time goes by. In such a dynamic environment, only using independent static sensor nodes, the change of the concentration cannot be tracked without the collaboration between different sensor nodes.
- It is difficult to track and monitor the active workers in a large-scale petrochemical plant. In a petrochemical plant, it is vitally important to identify the geographical locations of workers and to monitor the life signs (e.g., heart rate) of these workers when the leakage of toxic gases happens. The collaboration is necessary between different active workers to locate a worker and to estimate/predict the impact of the production environment on the health of the worker.
- For a certain sensor, it only can detect a toxic gas, and for a detecting system, different sensors are needed to detect different toxic gases. In the complex environment of a petrochemical plant, it is hard to make an optimal decision about what certain types of sensors are needed in a certain location to detect certain toxic gases. In addition, a petrochemical plant is an uncertain environment, and under this environment, a chemical reaction is possible between different toxic gases. This reaction produces new toxic gases that cannot be detected by the deployed sensors. Moreover, embedding all possible sensors into a detecting system is not cost-effective.
- It is difficult to set an optimal threshold for the sensed reading of toxic gas concentration. For example, for a carbon monoxide sensor, the predefined threshold is *x*, and in an accident, the leaking source of carbon monoxide gas is far away from this sensor. When the sensed reading of this sensor is larger than the predefined threshold *x*, the carbon monoxide gas has been widely diffused and has already gotten out of control.

Based on the characteristics of industrial problems, the CSI framework is designed and used to solve existing problems in industrial systems. It is based on analysing massive spatio-temporal data from various devices in IIoT environments.

**Figure 4:**
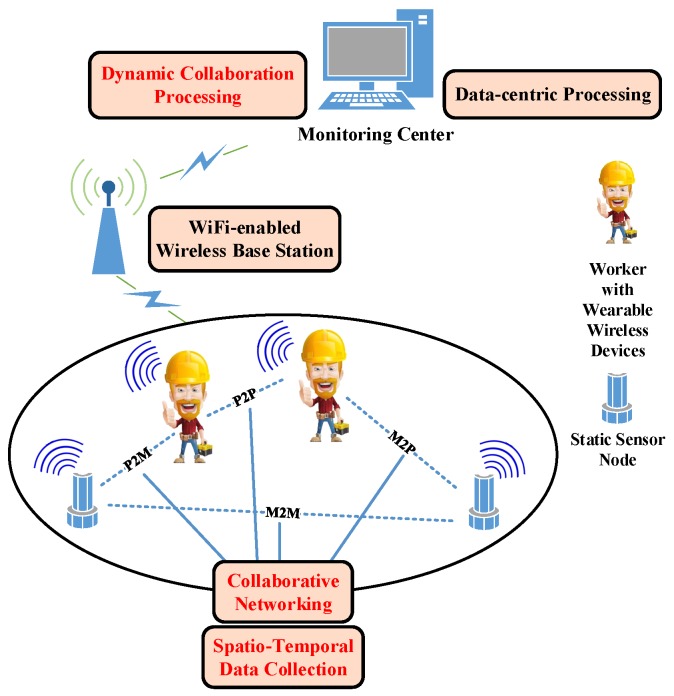
An application scenario of the CSI framework to improve the capability of detecting toxic gases in a large-scale petrochemical plant. This application consists of four components: sensor-embedded wearable wireless devices, static wireless sensor nodes, WiFi-enabled wireless base stations and a remote monitoring centre. The wearable wireless devices are worn by workers and collaborate with static wireless sensor nodes to sense the surrounding environment and collect spatio-temporal data. The data are sent to the remote monitoring centre via WiFi-enabled wireless base stations. In the monitoring centre, based on the collected data, by data-centric dynamic collaboration, the collaborative networking among different wireless devices can be achieved. Such networking constructs a problem-solving network to detect the leakage of toxic gases. Moreover, on such a networking basis, the CSI can be achieved in this scenario.

Figure 4 illustrates an on-going effort, a CSI-based system, which improves the capability of detecting toxic gases in a large-scale petrochemical plant.

**Figure 5:**
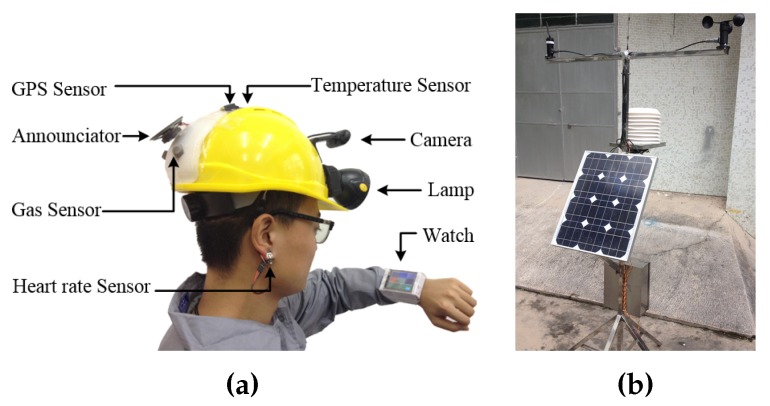
(**a**) Sensor-embedded wearable wireless devices: smart helmet and wrist watch; (**b**) static wireless sensor node. The smart helmet is sensor-embedded, and it works with the wrist watch to dynamically detect toxic gases. The static node is supported by solar energy and enables persistently measuring the concentration of gases in the air, e.g., CO, SO${}_{2}$ and CH${}_{4}$, and other environmental information, e.g., wind speed, humidity and temperature.

As the important two components of this application, Figure 5 provides the details of sensor-embedded wearable wireless devices and static wireless sensor nodes.

For example, first, along with the daily walking of workers in a petrochemical plant, massive spatio-temporal data are collected by smart helmets, and the smart helmets collaborate with static sensor nodes via communication-enabled wrist watches. Then, the collected data by smart helmets and static nodes are submitted to a remote monitoring centre. Finally, the massive spatio-temporal data are analysed based on the CSI framework. Such an analysis enables the collaborative networking among different wireless devices to construct a problem-solving network, and analysis results are returned to the wrist watches.

For the special problem, the leakage of toxic gases in large-scale petrochemical plants, because of the wide deployment of wireless devices, massive data are collected from these different devices. The collected data include different information from different locations and time points. Using the massive spatio-temporal data-based CSI framework, the widest detecting can be achieved as the efficient and cost-effective solution of the leakage problem.

#### 3.3.2. Citizen Sensing of La Poste

**Figure 6:**
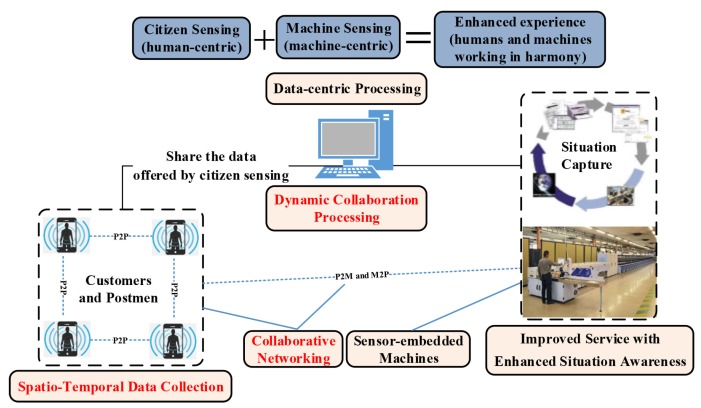
An example of networking two different data sources to improve the quality of service (a use case from La Poste). Integrating the spatio-temporal data from citizen sensing with the data from machine sensing provides enhanced experience and situational awareness. Such integration forms more complete information than either forms of sensing can provide alone. Additionally, it enables collaborative networking among different wireless devices.

Figure 6 provides an example: integrating two different data sources to improve the performance of services or solutions for mail delivery. This example is based on citizen sensing and machine sensing. Based on sensing and communication operations, sensors can share their data, which provides enhanced situational awareness that any system cannot offer alone.

From the example of Figure 6, the collaboration of different data sources can provide enhanced services or solutions with harmonious context. Such harmony can be achieved by the integration of data from different data sources, and the integration process is based on a certain logical sequence for these different data sources. Therefore, based on the integration capability of CSI for different data, the collaboration-based sensing intelligence improves the effectiveness of industrial systems to the resolution of complex problems.

Moreover, based on (i) the above discussion about the advances of CI and ISI and (ii) this on-going effort on CSI, a new application trend can be observed: realizing the interaction between the crowd wisdom of humans and sensing intelligence, in IIoT, for solving various complex problems of industry.

**Sensing intelligence of industrial applications interacting with the crowd wisdom of humans.** This includes two aspects: (i) participatory sensing in IIoT. Burke *et al.* assert: “participatory sensing will make deployed devices interactive, and participatory sensor networks enable different sensor-embedded machines to collect, analyse and mine data, and then to discover and share respective knowledge” [52]. In the era of big data, participatory sensing is the process where individuals and communities use devices or modules to collect and analyse systematic data for learning and discovering knowledge [53]. (ii) Crowd wisdom of humans: for example, as of March 2014, Twitter receives 500 million tweets per day, so mining the wisdom of crowds based on this type of big data has been made possible. To strengthen the decision-making ability of industrial systems, as an effective strategy, interacting with the crowd wisdom of humans has attracted the attention of researchers [54], and the strategy has the prospect of improving the ability of sensing intelligence [55].

In summary, the production/service of industry consists of a series of complex processes. High safety, efficiency and eco-friendliness are required during such production/service. However, how to make industrial environments and machines be safe and how to improve the efficiency of industrial production/service are long-term challenges. Meanwhile, the industrial production/service needs to ensure the friendly interaction with the surroundings. The data-centric collaboration uses comprehensive sensors and big data analytics to provide an efficient and cost-effective solution for a complex industrial problem.

## 4. Key Challenges and Open Issues

The CSI framework is used to face the growing demands of IIoT and to achieve the intelligence of industrial production/service. The key challenges and open issues on deploying this framework to practical industrial applications are worthy to be investigated and discussed, considering the characteristics of industrial problems under the background of IoT and big data analytics.

### 4.1. Key Challenges

The challenges come from these two aspects: data and functionality.

**Data:**

- Data analytics [56,57]: This is the bottleneck of the CSI framework, due to the lack of scalability for different datasets. Based on the characteristics of industrial problems, CSI analyses spatio-temporal datasets. These datasets are collected from different industrial equipment and different time points, and they have different semantics, different formats, different sizes and different contexts.
- Structuring data: Transforming unstructured data into a unified structured format for later analysis is a challenge for the CSI framework. As the basis of our intelligence framework, spatio-temporal data are not natively structured, e.g., daily running log data of different industrial equipment [58], and such unstructured data are typically text-heavy and contain important log information, such as dates, running parameters of equipment and values of these running parameters.
- Data privacy and knowledge access authorization [59,60]: Data privacy and knowledge access authorization are important for data owners. However, in the CSI framework, between data owners and data consumers, sharing data and knowledge is needed and important for good collaboration. For example, for two different industrial systems, they are data sources and they belong to different departments. Because of the high correlation of industrial processes, what level is just enough and how to define the level of privacy and access authorization between these two different industrial systems are challenges that are worth studying.
- Generic data model [61]: For making the spatio-temporal data of CSI framework be able to be used in knowledge discovery, a generic data model needs to be designed. However, different data have different formats, contexts, semantics, complexity and privacy requirements. The design of the generic data model is a challenge.

**Functionality:**

- Knowledge discovery [62]: In the era of big data, for mining the potential of big data analytics, it is vitally important to discover knowledge with understanding the nature (e.g., correlations, contexts and semantics) of data. However, it is still an open challenge for the CSI framework, because knowledge discovery is a complex process under the dynamic environment of industrial production/service.
- Effective and high-efficiency knowledge utilization [63]: Along with the wide use of sensors and wireless devices in IIoT, data are being produced by humans and machines at an unprecedented rate. This leads many industrial departments to explore the possibility of innovating with the data that are captured to be used as a part of future information and communication technology (ICT) services. The major challenge is how to release and use the knowledge that is mined from the massive data of industrial departments.
- Support for particular applications: In a particular application, specific data mining and training are required to perform knowledge discovery. For example, for detecting the leakage of toxic gases, based on static and wearable wireless node embedded sensors (they generate massive dynamic data: sensing records with time stamps and location tags), real-time data mining algorithms are needed to mine such data and to monitor dynamic industrial environments. The CSI framework is required to have the ability to support these special requirements and to make data owners and data consumers be able to communicate with each other for effective data mining and knowledge discovery.
- Real-time processing/controlling [64]. For example, because of the dynamic nature of industrial applications, real-time processing/controlling is necessary. However, due to the complexity of industrial processes and the differences of networking performance between different industrial devices, for an intelligence framework, real-time processing/controlling is hard to achieve.
- Interfaces between internal modules: The interfaces between different internal modules play the main role in affecting the performance of workflow. However, how to design effective interfaces is a challenge for the design of high-efficiency CSI framework. First, we need to make the inside of each internal module clear enough, and then, each internal module needs to provide respective parameters to design the corresponding interface. The difficulty of this design is: which parameters of each internal module affect workflow performance and how they affect it.
- Development of a security model [65]. A security model is capable of providing privacy and authority management. In the CSI framework, there are numerous roles and various corresponding parameters, e.g., data owners and data consumers. Therefore, how to design an appropriate and moderate security model is a challenge for achieving a safe and resource-shared intelligence framework.

### 4.2. Open Issues

Based on the aforementioned challenges, the open research issues are listed as follows, considering the particularity of the IIoT-based industry.

- Data integration [66]: Data are the basis of the CSI framework, and for the collaborative capability between different data sources, data integration is an important research issue. The goal of data integration is to combine the data residing at different sources and to tie these different sources controlled by different owners under a common schema. In the book [67], AnHai Doan *et al.* have provided and discussed: (i) the typical examples of data integration applications from different domains such as business, science and government; (ii) the goal of data integration and why it is a hard problem; and (iii) the data integration architecture. On this basis, considering the particularity of the IIoT-based industry, the biggest problem of data integration is how to automatically achieve a correct logical sequence for data integration, according to the real processes of industrial production/service.
- Data mining algorithms [68]: Based on the data collected from a variety of sensors and wireless devices that are distributed in industrial intelligent ecosystems, adequate data mining is an important issue for the CSI framework. Such mining is based on industrialized algorithms that are suitable for large-scale, complex and dynamic industrial production/service. For example, by mining the big monitoring data from a large-scale petrochemical plant, the potential leak sources of toxic gases can be predicted, and based on such a prediction, the safety of large-scale industrial production can be improved. The study in this topic is still very limited, due to the limitation of technology on big data analytics.
- Collaborative knowledge discovery algorithms [69]: For the CSI framework, designing algorithms to enable the collaboration between crowd wisdom and industrial sensing intelligence for discovering useful knowledge is a valuable research issue. However, due to the limitation of technology on the big data analytics and data processing in a large-scale, complex and dynamic industrial environment, as well as the problem of data integration, the study in collaborative knowledge discovery is still limited.
- Real-time algorithms [70]: Industrial production/service includes a series of dynamic processes. The real-time algorithms on data processing, data analysis and decision making are necessary for an intelligence framework to improve the timeliness of dynamic processes in industrial production/service. Shen Yin *et al.* [71] have proposed two real-time schemes for the fault-tolerant architecture proposed in [72]. This architecture is designed for the fault-tolerant control of industrial system. One is a gradient-based iterative tuning scheme for the real-time optimization of system performance. The other is an adaptive residual generator scheme for the real-time identification of the abnormal change of system parameters. Other than this fault-tolerant control, in other aspects of industry, real-time algorithms are very important, as well, for example detecting toxic gas in a highly dynamic production environment. However, there are no achievements for these “other aspects”.
- Trusted and privacy-protected model design [73]: The privacy of data and knowledge is important for data owners and data consumers in a collaborative framework. For the CSI framework, it is indispensable to study and design a trusted and privacy-protected (i) data model for data processing and analysis and (ii) a knowledge model for knowledge discovery and utilization. Such models are an important part of the collaborative framework. However, its design is based on different requirements from data owners and data consumers for different applications. There is no a unified standard for such a design.

## 5. Conclusions

Facing the growing demands of industrial production/service on improving the safety, efficiency and eco-friendliness, as well as meeting the cost-effective objectives, based on the IIoT and the characteristics of industrial problems, we have proposed the CSI framework combining CI and ISI. This sensing- and collaboration-based intelligence framework has the potential to improve the performance of industrial systems by providing better awareness and control to dynamic industrial environments and correlated production/service processes, with analysing and integrating massive spatio-temporal data. Moreover, because the spatio-temporal data are collected from things and humans, CSI can achieve improved automated decision making with ISI collaborating with the crowd wisdom of humans. In addition, the challenges and open issues for developing the CSI framework have been explored and discussed. The aim is to identify innovative research issues for industrial intelligence and to deploy the CSI framework to practical industrial applications.

## References

[1] Cheng C.W., Yao H.Q., Wu T.C. (2013). Applying data mining techniques to analyse the causes of major occupational accidents in the petrochemical industry. J. Loss Prev. Process Ind..

[2] Harris J., Sprott D., Torrance A., Shi M., Ranjan A., Sharma S., Biswas T., Sharma S., Kirsch P. Sharing industry knowledge to improve management of risks and safety in the use of explosives in surface mining. Proceedings of the 23rd World Mining Congress. Canadian Institute of Mining, Metallurgy and Petroleum.

[3] Bunse K., Vodicka M., Schönsleben P., Brülhart M., Ernst F.O. (2011). Integrating energy efficiency performance in production management–gap analysis between industrial needs and scientific literature. J. Clean. Product..

[4] Abbaszadeh S., Hassim M.H. (2014). Comparison of methods assessing environmental friendliness of petrochemical process design. J. Clean. Product..

[5] Chi Q., Yan H., Zhang C., Pang Z., Xu D.L. (2014). A reconfigurable smart sensor interface for industrial WSN in IoT environment. IEEE Trans. Ind. Informat..

[6] Gungor V.C., Hancke G.P. (2009). Industrial wireless sensor networks: Challenges, design principles, and technical approaches. IEEE Trans. Ind. Electron..

[7] Xu D.L., He W., Li S. (2014). Internet of things in industries: A survey. IEEE Trans. Ind. Inform..

[8] Vermesan O., Friess P. (2013). Internet of Things: Converging Technologies for Smart Environments and Integrated Ecosystems.

[9] Industrial Internet of Things. https://www.accenture.com/mz-en/technology-labs-insight-industrial-internet-of-things.aspx.

[10] Gebus S., Leiviskä K. (2009). Knowledge acquisition for decision support systems on an electronic assembly line. Expert Syst. Appl..

[11] Vyatkin V. (2013). Software engineering in industrial automation: State-of-the-art review. IEEE Trans. Ind. Inform..

[12] Gao Z., Saxen H., Gao C. (2013). Guest Editorial: Special section on data-driven approaches for complex industrial systems. IEEE Trans. Ind. Inform..

[13] Metzger M., Polakow G. (2011). A survey on applications of agent technology in industrial process control. IEEE Trans. Ind. Inform..

[14] Haas M.R. (2006). Knowledge gathering, team capabilities, and project performance in challenging work environments. Manag. Sci..

[15] Albrecht K. (2006). Social Intelligence: The New Science of Success.

[16] Devadasan P., Zhong H., Nof S.Y. (2013). Collaborative intelligence in knowledge based service planning. Expert Syst. Appl..

[17] Zheng V., Zheng Y., Xie X., Yang Q. (2012). Towards mobile intelligence: Learning from GPS history data for collaborative recommendation. Artif. Intell..

[18] Zheng V.W., Zheng Y., Xie X., Yang Q. Collaborative location and activity recommendations with gps history data. Proceedings of the 19th International Conference on World Wide Web.

[19] Zheng V., Cao B., Zheng Y., Xie X., Yang Q. (2010). Collaborative filtering meets mobile recommendation: A user-centred approach. AAAI.

[20] Leung K.W.T., Lee D.L., Lee W.C. CLR: a collaborative location recommendation framework based on co-clustering. Proceedings of the 34th International ACM SIGIR Conference on Research and Development in Information Retrieval.

[21] Cheng C., Yang H., Lyu M.R., King I. Where you like to go next: Successive point-of-interest recommendation. Proceedings of the Twenty-Third International Joint Conference on Artificial Intelligence.

[22] Bao J., Zheng Y., Wilkie D., Mokbel M.F. (2013). A survey on recommendations in location-based social networks. ACM Trans. Intell. Syst. Technol..

[23] Sattari M., Manguoglu M., Toroslu I.H., Symeonidis P., Senkul P., Manolopoulos Y. Geo-activity recommendations by using improved feature combination. Proceedings of the 2012 ACM Conference on Ubiquitous Computing.

[24] Lee W.H., Tseng S.S., Shieh W.Y. (2010). Collaborative real-time traffic information generation and sharing framework for the intelligent transportation system. Inform. Sci..

[25] Lee B.M., Ouyang J. (2014). Intelligent healthcare service by using collaborations between IoT personal health devices. Int. J. Bio-Sci. Bio-Technol..

[26] Pitt J., Bourazeri A., Nowak A., Roszczynska-Kurasinska M., Rychwalska A., Santiago I.R., Sanchez M.L., Florea M., Sanduleac M. (2013). Transforming big data into collective awareness. Computer.

[27] Baron R.S. (2005). So right it’s wrong: Groupthink and the ubiquitous nature of polarized group decision making. Adv. Exp. Soc. Psychol..

[28] Gardner M., Bieker J. Data mining solves tough semiconductor manufacturing problems. Proceedings of the Sixth ACM SIGKDD International Conference on Knowledge Discovery and Data Mining.

[29] Hackman J.R. (2011). Collaborative Intelligence: Using Teams to Solve Hard Problems.

[30] Argote L., Gruenfeld D., Naquin C. (2001). Group learning in organizations. Groups at Work: Theory and Research.

[31] Cai N., Gholami M., Yang L., Brennan R.W. (2012). Application-oriented intelligent middleware for distributed sensing and control. IEEE Trans. Syst. Man Cybernet. Part C: Appl. Rev..

[32] De Pellegrini F., Miorandi D., Vitturi S., Zanella A. (2006). On the use of wireless networks at low level of factory automation systems. IEEE Trans. Ind. Inform..

[33] Cao X., Chen J., Xiao Y., Sun Y. (2010). Building-environment control with wireless sensor and actuator networks: centralized versus distributed. IEEE Trans. Ind. Electron..

[34] Lee S.C., Jeon T.G., Hwang H.S., Kim C.S. (2007). Design and implementation of wireless sensor based-monitoring system for smart factory. Lect. Notes Comput. Sci..

[35] Korber H.J., Wattar H., Scholl G. (2007). Modular wireless real-time sensor/actuator network for factory automation applications. IEEE Trans. Ind. Inform..

[36] Tavner P., Xiang J., Spinato F. (2007). Reliability analysis for wind turbines. Wind Energy.

[37] Cecati C., Guinjoan F., Siano P., Spagnuolo G. (2013). Introduction to the special section on smart devices for renewable energy systems. IEEE Trans. Ind. Electron..

[38] Zhabelova G., Vyatkin V. (2012). Multiagent smart grid automation architecture based on IEC 61850/61499 intelligent logical nodes. IEEE Trans. Ind. Electron..

[39] Cecati C., Siano P. (2013). Special issue on advanced computational intelligence systems for smart grids planning and management. J. Ambient Intell. Hum. Comput..

[40] Byun J., Jeon B., Noh J., Kim Y., Park S. (2012). An intelligent self-adjusting sensor for smart home services based on ZigBee communications. IEEE Trans. Consum. Electron..

[41] Xia T., Xi L., Zhou X., Lee J. (2013). Condition-based maintenance for intelligent monitored series system with independent machine failure modes. Int. J. Product. Res..

[42] Cecílio J., Martins P., Costa J., Furtado P. A configurable middleware for processing in heterogeneous industrial intelligent sensors. Proceedings of the 2012 IEEE 16th International Conference on Intelligent Engineering Systems (INES).

[43] Wisniewski R., Svenstrup M., Pedersen A.S., Steiniche C.S. Certificate for safe emergency shutdown of wind turbines. Proceedings of the American Control Conference (ACC).

[44] Gungor V.C., Lu B., Hancke G.P. (2010). Opportunities and challenges of wireless sensor networks in smart grid. IEEE Trans. Ind. Electron..

[45] Ramchurn S.D., Vytelingum P., Rogers A., Jennings N.R. (2012). Putting the ”smarts” into the smart grid: A grand challenge for artificial intelligence. Commun. ACM.

[46] Castells M. (2014). Technopoles of the World: The Making of 21st Century Industrial Complexes.

[47] Lee K.H., Lee Y.J., Choi H., Chung Y.D., Moon B. (2012). Parallel data processing with MapReduce: A survey. AcM sIGMoD Rec..

[48] Martínez-López F.J., Casillas J. (2013). Artificial intelligence-based systems applied in industrial marketing: An historical overview, current and future insights. Ind. Market. Manag..

[49] Yu Q., Medioni G., Cohen I. Multiple target tracking using spatio-temporal markov chain monte carlo data association. Proceedings of the Conference on IEEE Computer Vision and Pattern Recognition, 2007, CVPR’07.

[50] Gilks W.R. (2005). Markov Chain Monte Carlo.

[51] Wang K., Lu H., Shu L., Rodrigues J.J. (2014). A context-aware system architecture for leak point detection in the large-scale petrochemical industry. IEEE Commun. Mag..

[52] Burke J., Estrin D., Hansen M., Parker A., Ramanathan N., Reddy S., Srivastava M. Participatory Sensing. Proceedings of the Workshop on World-Sensor-Web (WSW) at SenSys.

[53] Estrin D.L. Participatory sensing: Applications and architecture. Proceedings of the 8th International Conference on Mobile Systems, Applications, and Services (MobiSys).

[54] Guo B., Yu Z., Zhang D., Zhou X. (2014). From participatory sensing to mobile crowd sensing. CoRR.

[55] Guo B., Zhang D., Yu Z., Liang Y., Wang Z., Zhou X. (2013). From the internet of things to embedded intelligence. World Wide Web.

[56] Chen H., Chiang R.H., Storey V.C. (2012). Business intelligence and analytics: From big data to big impact. MIS Q..

[57] Bernecker T., Graf F., Kriegel H.P., Seiler N., Türmer C., Dill D. Knowing: A generic data analysis application. Proceedings of the 15th International Conference on Extending Database Technology.

[58] Bahga A., Madisetti V.K. (2012). Analyzing massive machine maintenance data in a computing cloud. IEEE Trans. Parallel Distribut. Syst..

[59] Li M., Lou W., Ren K. (2010). Data security and privacy in wireless body area networks. IEEE Wirel. Commun..

[60] Liu Q., Zhang X., Chen X., Wang L. (2014). The resource access authorization route problem in a collaborative manufacturing system. J. Intell. Manuf..

[61] Gómez L.I., Gómez S.A., Vaisman A.A. A generic data model and query language for spatiotemporal olap cube analysis. Proceedings of the 15th International Conference on Extending Database Technology.

[62] Van Der Aalst W. (2012). Process mining: Making knowledge discovery process centric. ACM SIGKDD Explor. Newslett..

[63] Dilling L., Lemos M.C. (2011). Creating usable science: Opportunities and constraints for climate knowledge use and their implications for science policy. Glob. Environ. Chang..

[64] Kopetz H. (2011). Real-Time Systems: Design Principles for Distributed Embedded Applications.

[65] Chau M., Li S.H., Urs S., Srinivasa S., Wang G.A. (2010). Intelligence and Security Informatics.

[66] Lenzerini M. Data integration: A theoretical perspective. Proceedings of the Twenty-First ACM SIGMOD-SIGACT-SIGART Symposium on Principles of Database Systems.

[67] Doan A., Halevy A., Ives Z. (2012). Principles of Data Integration.

[68] Larose D.T. (2014). Discovering Knowledge in Data: An Introduction to Data Mining.

[69] Atkinson M., Baxter R., Brezany P., Corcho O., Galea M., Parsons M., Snelling D., van Hemert J. (2013). The Data Bonanza: Improving Knowledge Discovery in Science, Engineering, and Business.

[70] Hatley D., Pirbhai I. (2013). Strategies for Real-Time System Specification.

[71] Yin S., Luo H., Ding S.X. (2014). Real-time implementation of fault-tolerant control systems with performance optimization. IEEE Trans. Ind. Electron..

[72] Ding S.X., Wang Y., Yin S., Zhang P., Yang Y., Ding E. (2012). Data-driven design of fault-tolerant control systems. Fault Detection, Supervision and Safety of Technical Processes.

[73] Bajaj S., Sion R. (2014). TrustedDB: A Trusted Hardware-Based Database with Privacy and Data Confidentiality. IEEE Trans. Knowl. Data Eng..

